# Using Smartphones to Help People with Intellectual and Sensory Disabilities Perform Daily Activities

**DOI:** 10.3389/fpubh.2017.00282

**Published:** 2017-10-24

**Authors:** Giulio E. Lancioni, Nirbhay N. Singh, Mark F. O’Reilly, Jeff Sigafoos, Gloria Alberti, Carmen Zimbaro, Valeria Chiariello

**Affiliations:** ^1^University of Bari, Bari, Italy; ^2^Medical College of Georgia, Augusta University, Augusta, GA, United States; ^3^University of Texas at Austin, Austin, TX, United States; ^4^Victoria University of Wellington, Wellington, New Zealand; ^5^Lega F. D’Oro Research Center, Osimo, Italy

**Keywords:** technology, smartphone, activities, intellectual disability, blindness, hearing impairment

## Abstract

**Background:**

People with mild-to-moderate intellectual disability and sensory impairments often fail to take initiative in starting and carrying out daily activities, with negative consequences for their occupational condition and social status. Their failure seems due to their inability to determine the right time for the activities and to remember all the activity steps.

**Aim:**

This study assessed a smartphone intervention, which was designed to help eight participants (four presenting with intellectual disability and blindness and four presenting with intellectual disability and hearing impairment) to independently start and carry out daily activities at appropriate times.

**Method:**

The intervention was introduced according to a non-concurrent multiple baseline design across participants. During the intervention, each participant was provided with a smartphone, which was fitted with the time schedule of his or her activities and the verbal or pictorial instructions for the single steps of those activities. When the time for an activity was reached, the participant was automatically reminded to start that activity and, thereafter, he or she was presented with the instructions for it.

**Results:**

The use of the smartphone intervention promoted great improvement over the baseline for all participants. That is, the participants managed to (a) independently start the activities at the scheduled times and (b) carry out those activities with high levels of accuracy.

**Conclusion:**

A smartphone intervention, such as that used in this study, may help people with mild-to-moderate intellectual disability and sensory impairments to successfully engage in daily activities.

## Introduction

People with mild-to-moderate intellectual disability and sensory impairments (i.e., blindness or hearing loss) may experience major difficulties engaging in functional daily activities independently (1–6). Indeed, they may be unable to determine the right time for the activities and fail to take initiative and start to perform them (7–9). Their situation can also be complicated by their apparent inability to remember all the steps of the activities and/or the steps’ correct sequence (10, 11). As a consequence of the aforementioned challenges, people with intellectual and sensory disabilities tend to be largely sedentary and passive with negative implications for their self-confidence, constructive engagement time, environmental sensory input, and social status (6, 12).

This negative perspective has created strong consensus on the need to find strategies to help them reach a more active and functional role within their daily contexts (11–15). It is also increasingly clear that aiming to enhance their activity engagement through extended staff assistance may not be feasible or desirable. In fact, staff resources are known to be generally limi-ted and probably insufficient to guarantee the necessary level of supervision. Moreover, an increase in the level of staff support/supervision would counter the people’s personal development in terms of self-determination, self-regulated engagement, and social image, and thus might prove detrimental (16–18).

In light of the above, efforts to increase people’s activity engagement have largely focused on providing them with activity support tools suited to their conditions (i.e., tools encompassing the step instructions of the activities to be performed) and teaching them to use those tools independently (15–20). The most basic tools consist of booklets with pictorial representations of objects related to the activity steps (i.e., with visual cues the people can use to help themselves remember the steps and their sequence) (20, 21). Other tools involve the use of technological devices (often modified for the purpose of the studies) such as (a) verbal recording devices that the people can use to obtain verbal instructions concerning the activity steps (5, 22–24), (b) simplified computer-aided systems, iPods, or video devices that the people can use to obtain static or dynamic visual instructions (i.e., pictures/photos or video clips illustrating the activity steps) (25–28), and (c) simplified computer-aided systems that automatically show (i.e., at preset time intervals) static or dynamic visual instructions for the activity steps (29).

Studies assessing the effectiveness of the aforementioned tools have reported encouraging results, that is, people appeared generally capable of using the tools to carry out multistep activities independently. It is noteworthy that, in contrast with the efforts to support the people’s independent performance of complex/functional activities, almost no attention has been paid to investigating whether they could also be helped to start those complex activities on their own, at the appropriate times (i.e., with a further enhancement of their active role) (30, 31).

In a recent, preliminary study aimed at pursuing both the aforementioned goals (i.e., enabling people to perform relevant multistep activities and also start those activities independently at the appropriate times), Lancioni et al. (32) compiled a technology-aided intervention relying on a smartphone and a tablet. The smartphone was set up to deliver timely reminders about the activities the participants were to carry out and the tablet served to present the participants the pictorial instructions for the steps of those activities. The results showed that all three participants managed to start the activities at the right times and carry them out correctly, thus suggesting that the intervention was suitable to achieve both target goals.

Notwithstanding the positive results of the study, caution is required in drawing conclusions given the small number of participants involved and the fact that the technology arrangement and instructions used would not be suitable for persons with blindness (33, 34). New research efforts to overcome these limitations and confirm the plausibility of targeting both goals (i.e., independent timely start and independent performance of relevant activities) are warranted. The present study was one such effort involving eight participants with intellectual disability, four presenting with blindness and the other four with hearing impairment. A smartphone intervention was used with each of them. In practice, each participant was provided with a smartphone, which was set up to (a) deliver verbal or vibratory and visual reminders at the times in which the activities were due and (b) present verbal or pictorial instructions for the single steps of those activities.

## Materials and Methods

### Participants

Table 1 reports the participants’ chronological ages and their Vineland age equivalences for receptive communication and personal and domestic daily living skills (35, 36). The participants, for whom pseudonyms are used, attended rehabilitation and care centers for persons with multiple disabilities and represented a convenience sample (37). They were divided into two groups, based on their sensory condition. Group 1 included the participants with total blindness (i.e., Sophie, Fergus, and Brady) or light/darkness discrimination (i.e., Nigel). Group 2 included the participants with severe hearing impairment and typical/functional sight (i.e., Owen, Karen, Loris, and Betty). The psychological records of the centers that the participants attended described their levels of intellectual disability to be in the mild/moderate or moderate ranges. Their Vineland age equivalences varied between 4 years and 3 months and 6 years and 6 months for receptive communication; between 3 years and 2 months and 4 years and 7 months for personal daily living skills; and between 4 years and 3 months and 7 years for domestic daily living skills (see Table 1).

**Table 1:** Participants’ chronological ages and Vineland age equivalences for receptive communication (RC) and Personal and Domestic Daily Living Skills (P/DLS and D/DLS).

Participants^a^	Chronological ages (years)	Vineland age equivalences^b^^,^^c^		
		RC	P/DLS	D/DLS
Sophie	18	4;3	3;2	4;3
Nigel	49	6;6	4;0	6;5
Fergus	43	6;6	3;10	6;5
Brady	45	6;2	3;11	4;7

Owen	25	5;1	4;7	7;0
Karen	57	5;10	4;7	6;9
Loris	19	4;8	3;6	6;5
Betty	32	5;6	4;7	7;0

*^a^The dotted line separates the participants of Group 1 and Group 2*.

*^b^Vineland age equivalences are reported in “years” (numbers before the semicolon) and “months” (numbers after the semicolon)*.

*^c^The age equivalences are based on the Italian standardization of the scales (35)*.

Staff and caregivers’ reports and direct observations had indicated that the participants had difficulties with daily activities (i.e., failing to remember the times at which they were due and the steps involved). In practice, participants tended to be dependent on external assistance. Staff and caregivers had expressed interest for a technology-aided approach that would encompass both reminders and verbal or pictorial instructions (i.e., to alert the participants about the activities to perform at the appropriate times and indicate the steps of those activities, respectively). Moreover, participants had shown willingness to use a smartphone such as that adopted in this study (i.e., after the functioning of that smartphone had been demonstrated to them by staff). In spite of this willingness, the participants were unable to give informed consent to the study. Thus, written informed consent was obtained from their legal representatives. The consent agreement allowed the legal representatives to withdraw the participants from the study at any time if they perceived the participants did not benefit from or were unhappy within the study. (None of the participants dropped out.) The study complied with the 1964 Helsinki declaration and its later amendments and was approved by the Ethics Committee of the Lega F. D’Oro, Osimo, Italy.

### Setting, Technology, and Activities

The study was conducted in the centers that the participants attended. The technology involved a Samsung Galaxy A3 smartphone with Android 5.1 Operating System, which included standard functions such as Bluetooth connection and Alarm and was fitted with the Easy Alarm YouTube application as well as with audio and video files. Audio files were used for the participants with visual impairment and typical hearing and consisted of the verbal reminders and instructions concerning the activity steps. There was one file for each of the activities included. The time for the performance of each of the activities was scheduled by the research assistant by linking the activity-related audio file with the alarm tone of the smartphone. As soon as such time was reached, the smartphone emitted a verbal reminder with the name of the activity to be performed. The reminder was then followed by each of the step instructions arranged for the activity. The research assistant scheduled the intervals between the reminder and the first activity instruction as well as between any pair of the following instructions of the sequence, based on preliminary observations of the participant’s performance speed. Longer intervals were scheduled following instructions related to more demanding steps, and vice-versa. The intervals could be readjusted in line with the participant’s progress. The instructions were conveyed directly *via* the smartphone that the participant carried with him or her or through a wireless Bluetooth earpiece that the participant wore during the sessions (thus avoiding to carry the smartphone).

Video files were used for the participants with hearing impairment and typical visual ability and consisted of static pictorial instructions, that is, photos of the object(s) involved in the single steps of the activities. As with the audio files, there was one video file for each activity included. The time for the performance of each activity was arranged through the Easy Alarm YouTube application. In essence, as the time for an activity was reached, the Easy Alarm YouTube application activated a reminder consisting of a vibratory signal and a general (preliminary/global) picture of the activity. This reminder was then automatically followed by the visual instructions for the single activity steps, which were separated by intervals scheduled according to the rules described for the verbal instructions. Again, the time intervals separating the instructions could be readjusted based on the participants’ progress. The participants carried the smartphone with them. The smartphone, which was hanging around their neck and reached their waist, was protected inside a transparent box (so the participants could handle it freely while watching the instructions without interfering with the preset functioning arrangements).

Pools of 10 or 12 daily activities of practical relevance were available for the participants (e.g., preparing coffee, setting the table for lunch, setting the table for recess, reordering the bathroom, reordering the bedroom, preparing material for the occupational room, putting away kitchen items, and preparing a service tray). The activities, which could vary across participants, included 20–25 steps. Table 2 lists the steps for one of those activities, that is, setting the table for recess. Six activities were scheduled for each session (i.e., a morning or afternoon period of 1.5–2 h). One or two daily sessions were typically available for the participants.

**Table 2:** Setting the table for recess.

1	Take the fruit from the refrigerator
2	Bring the fruit to the table
3	Take the instant coffee from the cupboard
4	Bring the coffee to the table
5	Take a bottle of water
6	Put the bottle on the table next to the water boiler
7	Take two cups
8	Put the cups on the table
9	Take the pitcher from the cupboard
10	Put the pitcher on the table
11	Take ice cubes from the freezer
12	Put the ice cubes into the pitcher
13	Take a bottle of lemonade
14	Put the bottle on the table next to the pitcher
15	Take two glasses
16	Put the glasses on the table
17	Take a dish with knifes and spoons
18	Put the dish on the table
19	Take the napkins from the cabinet
20	Put the napkins on the table
21	Tell the research assistant you are finished

### Research Assistants and Data Recording

Four college-graduate, research assistants experienced in the use of technology-aided programs with persons with multiple disabilities were in charge of the sessions across the different phases of the study, arranged the technology (smartphones) with verbal or vibratory and pictorial reminders and instructions, provided prompting in case of need, and carried out data recording (see below). Research assistants were involved in preliminary preparation meetings on each aspect of their role. Moreover, they were in communication among themselves during the study so as to clarify questions and ensure consistency across them.

Data recording concerned (a) the activities that the participants started correctly (i.e., at the appropriate time and independently) and (b) the activity steps they carried out correctly. A step was considered correct if the action required for it was performed independent of any prompting from the research assistant. Interrater agreement was checked in over 20% of the sessions (i.e., during which the research assistant and a reliability observer recorded the data) and was computed on groups of 10 activities for the first measure and single activities for the second measure. The percentages of agreement (which were determined by dividing the number of activities or steps with the same correct or incorrect score by the total number of activities or steps and multiplying by 100%) were in the 80–100 range, with means above 90 on both measures for all participants.

### Experimental Conditions and Data Analysis

The study was carried out according to a non-concurrent multiple baseline design across participants within each of the two groups of participants (38). Specifically, two baseline phases were implemented prior to the start of the intervention with the smartphone. Each of the two baseline phases included different numbers of sessions for the different participants of the groups. The number of baseline sessions for the single participants was preset. Yet, sessions would be added if the participants’ percentages of activities started correctly or activity steps carried out correctly were above 30 and the value of the last session exceeded those of previous sessions (this condition never applied). The intervention sessions served to determine the effects of the smartphone on each of the two measures. The baseline and intervention percentages of activities started correctly and activity steps carried out correctly were summarized/graphed as means per session over blocks of sessions, and their difference was analyzed *via* the “percentage of nonoverlapping data” (PND) method (39, 40).

#### Baseline I

Baseline I was to assess whether the participants started the activities correctly and included two to five sessions. The participant did not have any smartphone input and sat at a desk where conventional occupational material was available (e.g., jigsaws, family pictures, objects to be assembled, sorted or sanded, cardboards, and glue). Each session started with the participant receiving a list of the six activities scheduled for the session and the times at which the activities were due. The list consisted of (a) a paper sheet with small object replicas indicating the activities attached to the left column, and clock replicas with the times for the activities attached to the right column (Group 1) and (b) a paper sheet with the pictorial images of the activities on the left column and of clocks with the times for the activities on the right column (Group 2). A research assistant read those activities and times (Group 1) or pointed to those activities and times (Group 2) and ensured that the participant had the sheet in front of him or her.

#### Baseline II

Baseline II was to assess the participants’ level of correct activity performance (i.e., the number of activity steps that they performed correctly) and included three to five sessions. During each session, the research assistant asked the participant to carry out six acti-vities (i.e., one at a time). The research assistant provided verbal or physical prompting (encouragement) if the participant did not make any progress for about 1 min and corrected a step error if that precluded the adequate continuation of the activity. An activity would be interrupted after three consecutive prompting occasions, if the participant indicated that he or she did not know how to proceed. The steps not performed were scored incorrect. The participant would receive social approval for his or her efforts after each activity.

#### Intervention

The intervention phase was to assess the effects of the smartphone on the participants’ independent and timely start of the activities and correct performance of the activity steps and included 43–75 sessions. The participants had the smartphone with verbal instructions (Group 1) or pictorial instructions (Group 2), which worked as described in the *Setting, Technology, and Activities* section. Nigel and Brady (Group 1) received the verbal instruction *via* a wireless Bluetooth earpiece. Prior to the start of the intervention phase, the participants received five to seven practice sessions. During every practice session, the research assistant provided the participant with the verbal or physical prompting needed for an appropriate use of the technology (i.e., for responding to the smartphone’s reminders and following the smartphone’s activity instructions). During the regular intervention sessions that followed, the research assistant intervened with prompting if the participant did not respond to an activity reminder within about 30 s or made step errors during the performance of an activity that would interfere with its accurate completion. The participants received social approval after the performance of the activities (i.e., the last step instruction for each activity was to report to the research assistant; see Table 2) and at the end of the session.

## Results

**Figure 1:**
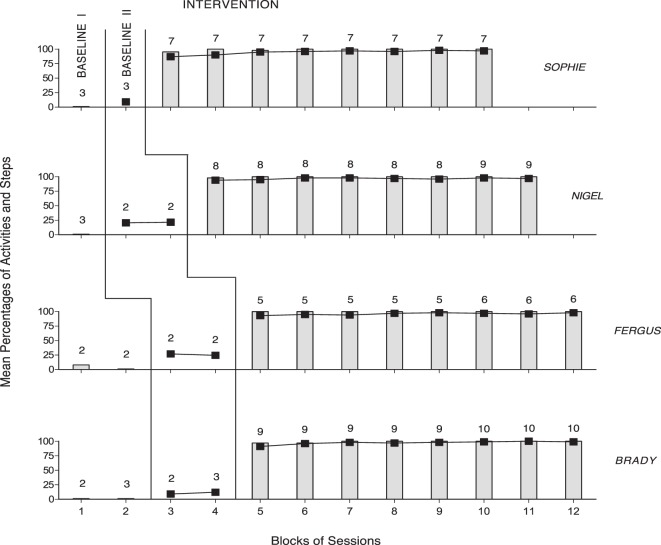
The four panels summarize the baseline and intervention data of the four members of Group 1 (i.e., Sophie, Nigel, Fergus, and Brady). The bars and black squares represent mean percentages of activities started correctly and mean percentages of activity steps carried out correctly per session, respectively, over blocks of sessions. The number of sessions included in the blocks is indicated by the numerals above the bars, squares, or bar–square combinations.

**Figure 2:**
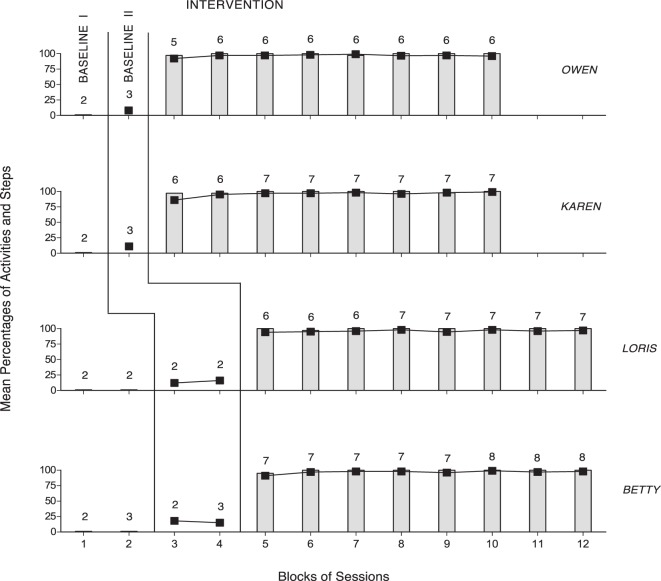
The four panels summarize the baseline and intervention data of the four members of Group 2 (i.e., Owen, Karen, Loris, and Betty). The data are plotted as in Figure 1.

The panels of Figures 1 and 2 summarize the baseline and intervention data of the four members of Group 1 and the four members of Group 2, respectively. During Baseline I, the participants’ mean percentages of activities started correctly were 0 or close to 0. During Baseline II, the participants’ mean percentages of activity steps carried out correctly were below 30. During the intervention phase (i.e., following the five to seven practice sessions that are not reported in the figures), the participants’ mean percentages of activities started correctly per session were (nearly) 100. That is, the participants responded to all smartphone-regulated activity reminders or missed only very few of them (i.e., five or less in total). The mean percentages of correct activity steps per session (i.e., across all the activities available within the session) increased to near or above 95 for all participants, with no apparent difference between Group 1 and Group 2. Comparisons of the intervention with the Baseline I and Baseline II session data on correctly started activities and correct activity steps, according to the PND method, showed indices of 1.0 for all participants (i.e., all their intervention data points on each measure exceeded their baseline levels).

## Discussion

The results of this study indicate that the smartphone intervention was effective in helping both groups of participants to correctly start and carry out the activities scheduled during the sessions. Indeed, all participants seemed to respond successfully to the reminders and activity instructions presented by the smartphone, thus showing clear performance improvement compared to the baseline periods. Moreover, the participants seemed to enjoy the sessions and their activity management (i.e., start and execution) with the support of the smartphone, as indicated by a number of informal reports, which underlined their eagerness to be involved in the sessions and their satisfaction with their activity engagement. In light of the above, a number of considerations may be in order.

First, these data confirm preliminary, pilot findings and show that persons with mild and moderate intellectual disabilities and sensory impairments can manage the independent and timely start and correct performance of relevant activities through the support of technology (32). Although caution is needed in drawing general conclusions given the relatively small number of participants involved in this study, one could still argue that the data reported here add considerably to the evidence previously available (33, 34). Indeed, these data might be taken as a new, encouraging reference for education and rehabilitation contexts in charge of people like the participants of this study.

Second, the smartphone intervention allowed one to arrange verbal stimuli or combinations of vibratory and visual stimuli as activity reminders. Those reminders, which were deemed suitable for individuals with blindness and hearing impairment, respectively, proved highly effective with the two groups of participants involved in this study. Similarly, the smartphone could be fitted with audio or visual files and thus serve as an effective instruction tool for all participants irrespective of their type of sensory impairment. This versatility of the technology, and its accessibility (commercial availability) and affordability can be considered great practical advantages that may enable education and rehabilitation contexts to successfully set up intervention programs for persons with different requirements.

Third, in addition to being flexible and affordable, the smartphone intervention is also practical to use for participants and staff. Participants who rely on verbal reminders and verbal instructions may not need to carry the smartphone with them. It is sufficient that they wear a wireless Bluetooth earpiece during the sessions (as it was done by two participants in this study). Participants with hearing impairment need to carry the smartphone with them. Carrying it enables them to readily perceive the reminders and see the visual instructions. The smartphone can be hanging around their neck or can be attached to their belt, in line with their preference. Staff can easily modify audio or video files in terms of content or time intervals and thus can make intervention adjustments in relation to participants’ general skills and progress.

Fourth, successful performance with the smartphone intervention probably increases the participants’ levels of self-confidence and satisfaction (41, 42). These aspects may contribute (together with the social approval following the performance of the activities) to ensure maintenance of positive activity engagement (43, 44). The participants’ new performance skills might also be seen as instrumental in facilitating a higher level of approval and appreciation from their education/rehabilitation and social context with potentially beneficial consequences for their mood and overall quality of life (45–47). While all these statements appear quite reasonable in light of previous literature and informal observations, research still needs to address them directly to determine their accuracy (42, 45).

Fifth, a main limitation of the study is the relatively small number of participants involved. Obviously, new studies with additional participants are required to verify the suitability of the smartphone intervention, the reliability of the findings, as well as the maintenance and generalization of the activity skills (33, 34). A second limitation is the lack of a social validation assessment aimed at determining the opinion of staff personnel about the smartphone’s impact and usability within everyday contexts (48, 49). Such an opinion might significantly add to the data and partly predict the future adoption of smartphones within those contexts (50, 51). Another apparent limitation is the lack of reliability (procedural fidelity) checks on the research assistants’ performance. In this study, research assistants’ experience and preliminary preparation were considered the best guarantee of procedural fidelity. Notwithstanding the directness of this view, the use of reliability checks remains a basic methodological requirement (52).

In conclusion, the results indicate that the smartphone intervention was suitable to support correct start and accurate performance of daily activities by persons with intellectual and sensory disabilities. Before general statements can be made about the usability of smartphones, new research would need to address the main limitations of the present study and determine the dependability of the results reported. New research efforts may also focus on (a) gathering formal evidence about participants’ satisfaction with the intervention conditions and their performance (i.e., by recording their preferences or indices of happiness) (53, 54) and (b) determining the overall acceptability of smartphones within everyday contexts (51).

## Ethics Statement

Appropriate institutional board approval and written informed consent were obtained for the study. All procedures performed were in accordance with the ethical standards of the institutional and/or national research committee and with the 1964 Helsinki Declaration and its later amendments or comparable ethical standards.

## Author Contributions

GL, NS, MO, and JS were responsible for setting up the study, acquiring/analyzing the data, and writing/editing the manuscript. GA, CZ, and VC contributed in acquiring and analyzing the data and editing the manuscript.

## Conflict of Interest Statement

The authors declare that the research was conducted in the absence of any commercial or financial relationships that could be construed as a potential conflict of interest.
